# Detection and Comparison of Sow Serum Samples from Herds Regularly Mass Vaccinated with Porcine Reproductive and Respiratory Syndrome Modified Live Virus Using Four Commercial Enzyme-Linked Immunosorbent Assays and Neutralizing Tests

**DOI:** 10.3390/vetsci12050502

**Published:** 2025-05-20

**Authors:** Chaosi Li, Gang Wang, Zhicheng Liu, Shuhe Fang, Aihua Fan, Kai Chen, Jianfeng Zhang

**Affiliations:** 1 Boehringer Ingelheim Animal Health (Shanghai) Co., Ltd., Shanghai 200040, China; lichaosi14@163.com (C.L.); brook.fang@boehringer-ingelheim.com (S.F.); fiona.fan@boehringer-ingelheim.com (A.F.); 2 Guangdong Province Key Laboratory of Livestock Disease Prevention, Institute of Animal Health, Guangdong Academy of Agricultural Sciences, Guangzhou 510640, China; rainman136@aliyun.com; 3 Shandong Provincial Key Laboratory of Zoonoses, College of Veterinary Medicine, Shandong Agricultural University, Tai’an 271000, China; wg0381@163.com

**Keywords:** porcine reproductive and respiratory syndrome virus, enzyme-linked immunosorbent assay, neutralizing antibodies, mass vaccination, modified live virus

## Abstract

Porcine reproductive and respiratory syndrome virus (PRRSV) modified live virus (MLV) vaccination is used to control PRRSV. In China, farms conduct random sampling from sow herds every 4 to 6 months. They use the enzyme-linked immunosorbent assay (ELISA) method to monitor the immune status of the herd by tracking the positive rate or the sample-to-positive ratio. However, in farms that implement mass vaccination and have stable production, the positive rate of ELISA antibodies has decreased, especially in high-parity sows. This poses a considerable challenge to the current monitoring approach of PRRSV immunity. It remains unclear whether this reflects insufficient sensitivity of the kits for these special scenarios or the fact that the sows have truly lost immunity. In this study, 233 samples from four farms (A–D) across different regions of China were acquired. They were tested using four representative ELISA kits, two targeting the nucleocapsid protein (N) and two targeting the glycoprotein (GP) to evaluate PRRS immune status. The respective sample positive rates in A–D were 57.1–100%, 50.9–100%, 50–100%, and 75.7–100% using the kits. The positive rates using the four ELISA kits were 50.0–75.7%, 70.0–75.7%, 82.5–97.1%, and 100%, respectively, with poor agreement among them. The positive rates and humoral antibody levels for parity 1 and 2 sows were significantly lower than those with higher parities (>4). Eighty-eight ELISA-negative samples identified using ELISA kit A were verified using a viral neutralizing test (VNT), with only 15.9% of the samples testing negative. In conclusion, the ELISA antibody negativity issue existed, mostly occurring in specific farms tested using a specific kit. However, the low correlation with the VNT results and the poor agreements among the kits suggest that relying on one ELISA test is insufficient to monitor the immune status of PRRSV MLV-vaccinated herds.

## Simple Summary

The immune status of porcine reproductive and respiratory syndrome (PRRS), evaluated and monitored using one specific commercial enzyme-linked immunosorbent assay (ELISA) kit, was well received and widely implemented by pig farmers. However, ELISA antibody-negative cases were frequently reported in sow herds regularly mass vaccinated with PRRS virus modified live virus (PRRSV MLV). In this study, a cross-sectional investigation was conducted to assess four main issues: firstly, whether ELISA-negative cases commonly exist in MLV-vaccinated sows; secondly, to assess the correlation between common commercial ELISA kits based on the nucleocapsid (N)-protein and glycoprotein (GP)-protein, including the positive rate, antibody level, coefficient of variation, and agreement rate; thirdly, to investigate if there are disparities within sows at different parities; and lastly, to determine if the sole use of N-protein-based ELISA kits is adequate to evaluate the immune response following vaccination.

## 1. Introduction

Porcine reproductive and respiratory syndrome virus (PRRSV) is a single-strand RNA virus with high diversity because of its tendency to mutate and is generally divided into two genotypes: European type 1 and North American type 2 [1,2]. In 2006, a highly pathogenic strain of PRRSV (NADC-30) emerged in China [3], followed by the identification and spread of NADC-30-like strains since 2014 [4,5].

Porcine reproductive and respiratory syndrome (PRRS) is regarded as one of the most devastating swine diseases. It causes large economic losses in the pig industry worldwide, including reproductive failure in sows, extended non-reproductive days, increased culling and death, and reduced growth and feed efficiency in growing and finishing herds [6,7,8].

PRRSV modified live virus (PRRSV MLV) vaccination combined with biosecurity remains the major PRRSV control strategy for most Chinese commercial farms. PRRSV MLV vaccines can confer complete homologous protection and partial heterogeneous protection, thereby reducing economic losses during PRRSV infection [9,10,11]. In China, most PRRSV-positive sow farms would carry out three to four mass vaccinations per year. Routine monitoring of PRRSV antibody levels to evaluate vaccination compliance or immunity status is commonly undertaken.

There are dozens of available commercial PRRSV enzyme-linked immunosorbent assay (ELISA) kits on the market, which are manufactured by multinational corporations and local companies. The sensitivity and specificity of these kits vary depending on studies and scenarios. In 2018, Biernacka et al. [9] assessed six commercial PRRSV ELISA kits. Under diverse scenarios, such as in pig herds with early infection, the disparities in performance seemed to be practically insignificant. Nevertheless, in positive and stable herds, some kits yielded results that did not mirror the infection status in specific age groups [9]. In 2020, Díaz et al. employed four commercial ELISA kits to assess the PRRSV levels before and after immunization. The application of ELISA to monitor multiple PRRS MLV-vaccinated sows was highly restricted due to the variability of the humoral responses and the moderate consistency between tests [10]. In 2022, Fiers et al. employed the IDEXX PRRS X3 Ab test and CIVTEST SUIS PRRS E/S test and discovered that there were non-responders among multi-vaccinated sows. The overall proportion of ELISA non-responders was relatively low (3.5–4.1%), yet the proportion of herds with ELISA non-responders was rather high (40%), indicating that this phenomenon was not confined to specific herds [12].

However, field veterinarians often report lower than expected PRRSV ELISA-positive rates in farms with good compliance, in which, theoretically, all the samples should be positive. Herein, a cross-sectional study was performed in four Chinese large-scale farrow to wean farms located in four separate areas that conduct routine PRRSV MLV vaccination. We aimed to assess four main issues: firstly, if the ELISA-negative issue commonly exists in PRRSV MLV mass vaccinated herds; secondly, to evaluate the correlation and agreement between commonly used nucleocapsid (N)-protein-based and glycoprotein (GP)-protein-based commercial ELISA kits in China regarding detection outcomes and values; thirdly, to investigate whether there are differences in ELISA-positive rates and values among sows of different parities within regularly MLV-vaccinated sow herds with PRRS status 1-B; and lastly, to determine if using only N-protein-based ELISA kits is sufficient to evaluate the immune effect after vaccination.

## 2. Materials and Methods

### 2.1. Investigated Farms

The samples were provided voluntarily by the owners of four farms for testing and further study. The sow farms (with herd sizes ranging between 1000 and 5000 heads) were located in four provinces of China: Northeastern Liaoning, Northwestern Gansu, Southeastern China’s Fujian, and Central Jiangxi. All farms raised Landrace × Large White crossbred sows. Despite routine PRRSV MLV vaccinations being regularly and properly performed, all four farms have reported issues with PRRSV ELISA antibody negativity. The immunization strategy for these farms was as follows: gilts received two full-dose vaccinations (Ingelvac PRRS^®^ MLV, Boehringer, Ingelheim, Germany) between 120 days of age and before breeding. Meanwhile, the sow herd received 3–4 full-dose vaccinations annually. Blood samples for the experiment were collected at least 21 days after the gilts had received their second MLV vaccination. The PRRS status in these four farms ranged from 1-B to 2Vx, according to the American Association of Swine Veterinarians (AASV) PRRS Classification [13].

### 2.2. Experimental Design

The number of samples collected was computed based on the implementation of an individual farm. The specific calculation approach was as follows. In accordance with the customer’s expectation of the positive rate of PRRSV antibodies (>90%), these farms contained no more than 5000 productive sows. Using a 95% confidence interval, we calculated that 30 samples needed to be collected from each farm [14]. If all 30 samples were positive, then the negative rate was no more than 10%. If at least one sample was negative, then the positive rate was less than 90%, and the antibody-positive level failed to meet the standard. Nevertheless, in field practice, the specific number of samples provided by the farm may vary, but all four provided more than 30 samples. The impact of African Swine Fever (ASF) prevention measures, such as temporarily suspended production or partial depopulation, meant that the parity structures of these herds differed from typical scenarios. When taking serological samples, this study adhered to each farm’s disease control protocols while aiming to represent the parity distribution across the farms. A total of 233 serum samples were obtained with cross-sectional random sampling from the replacement gilt and sow populations (farm A: 70 samples, farm B: 53 samples, farm C: 40 samples, and farm D: 70 samples). The specific parity (P) sample distributions are shown in Table 1. For clarity, gilts that bred but had not farrowed were classified as P0.

### 2.3. ELISA Tests

Four representative commercial PRRSV ELISA kits that are widely used on the Chinese market were selected for testing in this study. These included two N-protein-based ELISA kits: Kit A (IDEXX PRRS X3 Ab ELISA (IDEXX Laboratories, Westbrook, ME, USA)) and Kit B (AB ELISA Kit (JNT, Beijing, China)), as well as two GP-protein-based kits: Kit C (PRRS ELISA kit (ANHEAL, Beijing, China)) and Kit D (Civtest Suis E/S (HIPRA, Amer, Spain)). All 233 serum samples were tested using these four kits, conducted strictly according to the manufacturers’ instructions. The results from Kits A and B are expressed as sample-to-positive (S/P) ratios, with a cutoff value greater than 0.4 indicating a positive result. The results for Kit C are reported as optical density at 450 nm (OD450 nm) absorbance values, for which readings greater than 0.3 times the OD450 nm value of the positive control were considered positive. The results for Kit D are presented as the Relative Index Percentage (IRPC), with a threshold greater than 20 for positivity.

### 2.4. Serum Virus Neutralization Test (VNT)

In total, 88 samples that tested negative using Kit A were chosen for a VNT. PRRSV strain VR2332 (GenBank Accession no EF536003.1) was chosen as the neutralized virus in the test, which was performed as described previously [15]. Briefly, serum samples were heat-inactivated at 56 °C for 30 min. Then, 125 µL of serum was 2-fold serially diluted with 125 µL of Minimal Essential Medium (Gibco, Beijing, China) with antibiotics (0.1% Penylstreptomycin) from 1:2 to 1:256 before being mixed with an equal volume of VR2332 containing 100 50% tissue culture infectious dose (TCID50). Each mixture was transferred to monolayers of Marc-145 cells (PRRSV permissive cells) in 96-well plates after incubation for 1 h at 37 °C in an incubator containing 5% CO_2_. Each dilution was added to four wells as replications. Cells were then examined for cytopathic effects (CPEs). The CPE was used to determine the end point titers, which were calculated as the reciprocal of the highest serum dilution required to neutralize 200 TCID50 of PRRSV in 90% of the wells [16,17]. Samples with a VN titer ≥ 2 Log2 (4) were considered to be seropositive in the VNT [12].

### 2.5. Statistical Analysis

In this study, statistical analyses were conducted using GraphPad Prism v10 (GraphPad Inc., La Jolla, CA, USA), SPSS v27 (IBM Corp., Armonk, NY, USA), and Excel 2023 (Microsoft, Redmond, WA, USA). Specifically, basic statistics and chart creation were performed using Microsoft Excel. A normal distribution test was conducted before significance analysis. Mean differences for the S/P values of the total samples from Kits A, B, and C; antibody-positive rates of the four farms; and S/P or IRPC values across parity groups of the total samples were carried out using the Kruskal–Wallis test, followed by Dunn’s multiple comparisons test, in GraphPad Prism v10. For correlation analyses, linear relationships between paired values from every two ELISA kits, the ELISA results, and the VNT results were first assessed by evaluating the normal distribution of these data, followed by calculating the Spearman correlation coefficient (r) using GraphPad Prism v10. Kappa analysis was then conducted to evaluate the agreement on positivity and negativity between every two kits using Microsoft Excel. A Kappa value above 0.75 was considered to indicate high agreement, values between 0.4 and 0.75 indicated medium agreement, and values below 0.4 were considered to indicate low agreement. Chi-squared tests were used to compare positive rates across parities of total samples in SPSS v27. The result was considered a significant difference when the *p*-value was below 0.05. An R value greater than 0.79 was considered to indicate a strong correlation, 0.5–0.79 indicated a moderate correlation, 0–0.5 suggested a weak positive or negative association, and 0 indicated no linear or monotonic relationship between the two variables. A Kappa value greater than 0.8 indicated very strong agreement, 0.6–0.79 indicated strong agreement, 0.4–0.59 indicated moderate agreement, 0.2–0.39 indicated weak agreement, and a value less than 0.2 indicated no agreement. This combination of methods ensured a comprehensive evaluation of the test results.

## 3. Results

### 3.1. The ELISA Positive Rate and Value in the Four Farms

Positive rates and mean values were used to evaluate the immune status of the four commercial farms across all the samples, as illustrated in Table 2. To standardize the comparison, Kit D’s IRPC values were adjusted by dividing them by 100.

The positive rates of Farm A from the four kits were Kit A, 57.1%; Kit B, 75.7; Kit C, 80.0%; and Kit D, 100.0%. Farm B’s positive rates were Kit A, 50.9%; Kit B, 73.6%; Kit C, 94.3%; and Kit D, 100.0%. Farm C’s positive rates were Kit A, 50.0%; Kit B, 70.0%; Kit C, 82.5%; and Kit D, 100.0%. Farm D’s positive rates were Kit A, 75.7%; Kit B, 75.7%; Kit, C 97.1%; and Kit D, 100.0%. Farm C showed the lowest positive rate with Kits A and B, and farm A had the lowest positive with Kit C. However, these kits produced consistent results for farm D, presenting the highest positive rate compared to the other three farms (Table 2).

The ELISA test result averages for Farm A were Kit A, 0.51; Kit B, 0.66; Kit C, 0.59; and Kit D, 215.1. The averages for Farm B were Kit A, 0.59; Kit B, 0.77; Kit C, 0.92, and Kit D, 216.2. The averages for Farm C were Kit A, 0.46; Kit B, 0.93; Kit C, 0.68; and Kit D, 238. The averages for Farm D were Kit A, 0.80; Kit B, 0.65; Kit C, 0.77; and Kit D, 142.4. The S/P average of the four farms ranked differently using the different kits. Farm D had the highest ELISA value average with Kit A, farm C with Kit B and Kit D, and farm B with Kit C (Table 2).

As a rule of thumb, according to the farms’ own principles, a positive rate below 60% was regarded as an unqualified immune response. In this regard, farms A, B, and C were regarded as unqualified for Kit A, accounting for 75%. However, none of the farms were unqualified for Kits B, C, and D.

### 3.2. Statistical Summary of the ELISA Results

**Figure 1:**
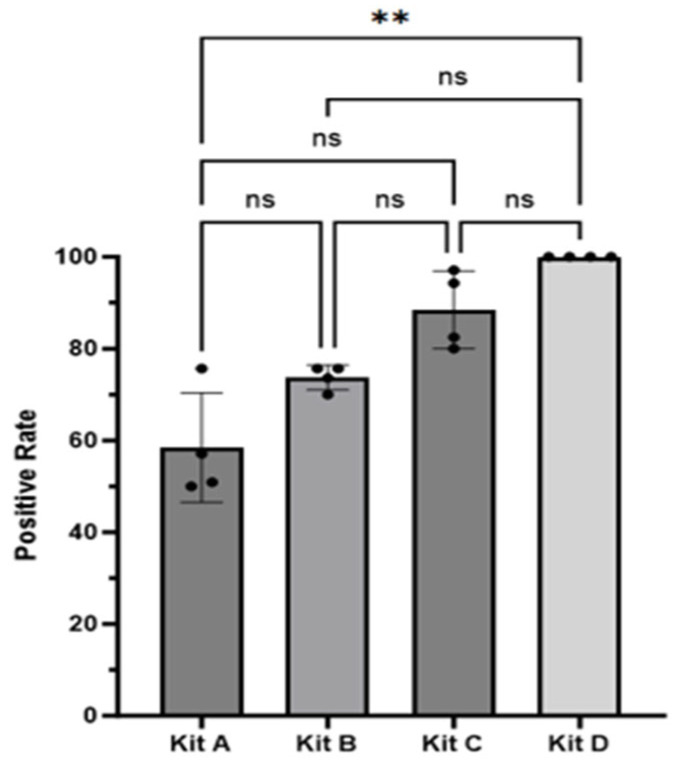
Comparison of the positive rates among the four ELISA kits. Analysis of the positive rate using the Kruskal–Wallis test was conducted, followed by Dunn’s test, with each farm treated as an observational unit. A significant difference was observed between Kit A and Kit D (*p* = 0.04), while no significant difference was found between the other pairs of kits (** *p* > 0.01). ns, not statistically significant; ELISA, enzyme-linked immunosorbent assay.

In this section, the four commercial ELISA kits were compared by their positive rates and average value. The positive rates of these four kits across the total samples were as follows: D (100.0%) > C (88.8%) > B (74.2%) > A (60.1%) (Table 2). Taking each pig farm as an observation unit, the positive rate range of Kit A was 50.0% to 75.7%, Kit B was 70.0% to 75.7%, and Kit C was 82.5% to 97.1%(Table 2). Kit D demonstrated a 100% positive rate in all four pig farms tested. Statistical analyses revealed that Kit A’s positive rate was significantly lower than that of Kit D (*p* = 0.04). Even though differences were observed among Kits A, B, and C, they did not reach statistical significance (*p* > 0.05) (Figure 1).

**Figure 2:**
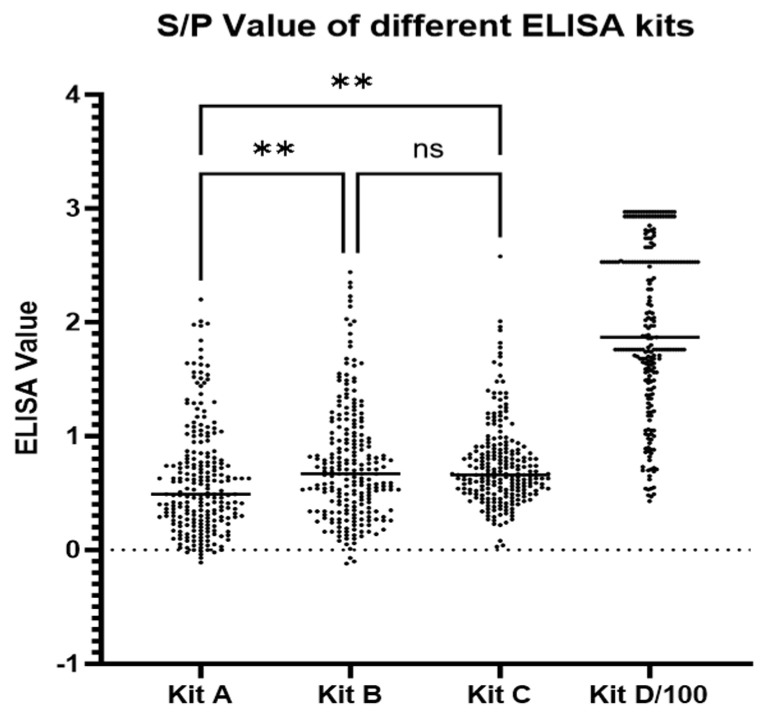
Test results distribution of the different ELISA kits. The IRPC values for Kit D were normalized when divided by 100. The results from all four kits show a dispersed distribution. ** *p* < 0.01. ns, not statistically significant. S/P, sample-to-positive ratio; IRPC, Relative Index Percentage.

The average S/P or IRPC value for the total samples from the four kits were all positive according to each kit’s criteria, though with wide ranges. For Kit A, it was 0.61 (range −0.11, to 2.20). For Kit B, it was 0.76 (range −0.12 to 2.44). For Kit C, it was 0.75 (range 0.03 to 2.58). For Kit D, it was 197.45 (range 43.05 to 253.30). The Kruskal–Wallis test of the S/P values showed that Kit A had a significantly lower mean value than Kits B and C (*p* < 0.01), with no significant difference between Kits B and C (Figure 2). All kits exhibited considerable variability in their detection values. The distribution patterns of Kits A, B, and C were comparable, whereas Kit D’s IRPC values were more concentrated at the higher end of the spectrum. Notably, Kit D also demonstrated the lowest coefficient of variation (CV) at 37% (Table 2).

### 3.3. Correlation and Agreement Analysis of the Four ELISA Kits

**Figure 3:**
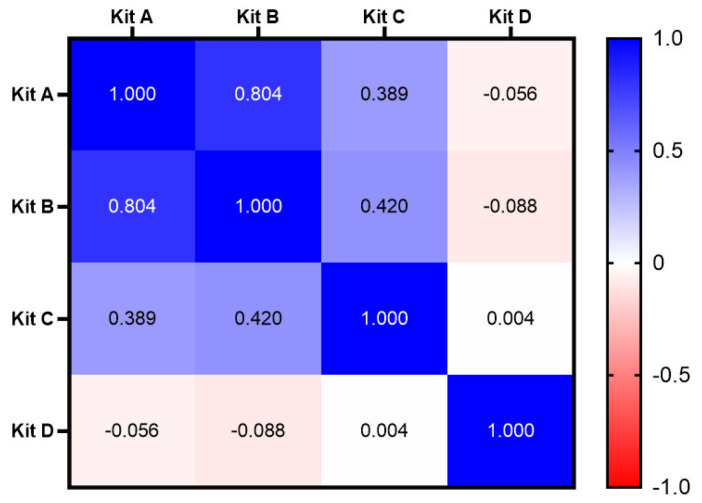
**Correlation matrix of the test values from the four ELISA kits.** Kits A and B showed a strong correlation (r = 0.804, *p* < 0.001). Moderate correlations existed between Kits A and C (r = 0.389, *p* < 0.001) and Kits B and C (r = 0.420, *p* < 0.001). Kit D had no correlations with any of the other kits.

Spearman correlation coefficient (r) analysis was conducted with the paired ELISA test values of the four diagnostic kits (Figure 3). Kappa agreement analysis was calculated using the positivity/negativity results of these kits (Table 3).

The correlation analysis revealed a strong correlation between Kits A and B (r = 0.80; *p* = 0.00). Weak correlations were observed between Kits A and C (r = 0.39) and Kits B and C (r = 0.42) (both *p* = 0.00). There was no correlation between Kits A and D (r = −0.06), Kits B and D (r = −0.09), and Kits C and D (r = 0.00), with *p*-values of 0.39, 0.18, and 0.95, respectively (Figure 3).

Kappa analysis demonstrated moderate agreement between Kits A and B (Kappa = 0.59), while no agreement was observed between Kits A and C and Kits B and C (Kappa < 0.20). Kit D showed uniformly positive results, and as such, it exhibited no agreement with the other three kits (Table 3).

### 3.4. Comparison of Antibody-Positive Rates and Mean Values Across Different Parity Groups

**Figure 4:**
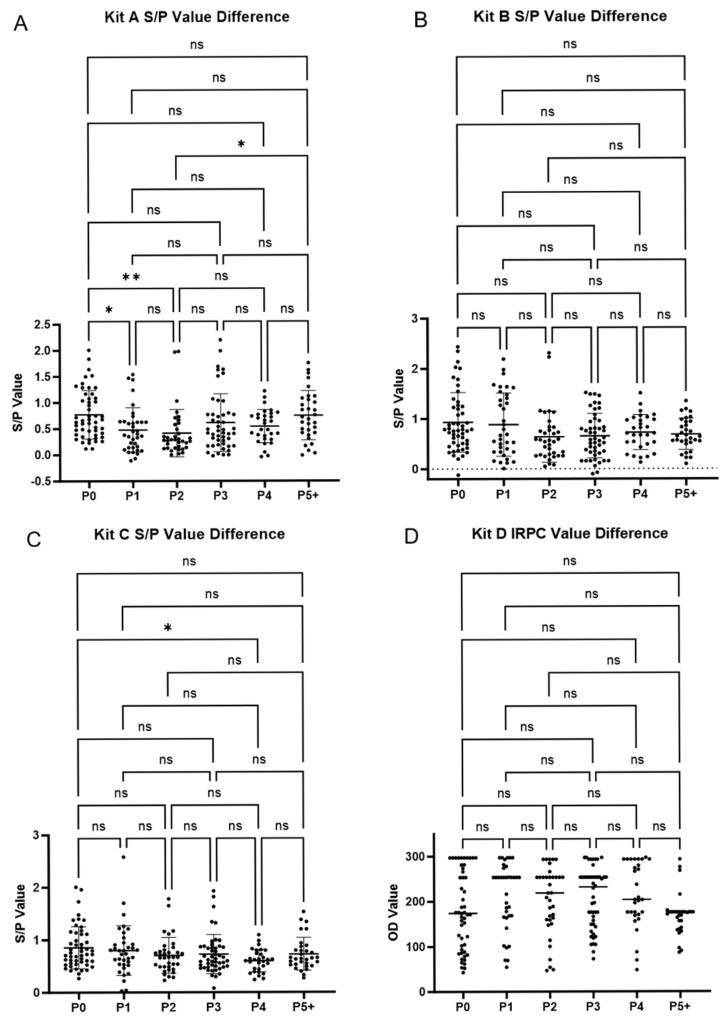
Comparison of the mean ELISA antibody values across different parities. (**A**–**D**) The Kruskal–Wallis test and Dunn’s multiple comparisons test results of the mean ELISA antibody values of the four kits, each based on a total of 233 samples. The horizontal bar of each parity group (P0 to P5+) represents the mean value of that group, while the vertical error bars represent the standard deviation (SD). Significance levels are indicated as follows: * *p* < 0.05 and ** *p* < 0.01. ns, not statistically significant.

Statistical analyses of antibody-positive rates and mean values across different parity groups were conducted (Table 4 and Figure 4A–D). The varying parity structures across the different pig farms (Table 1) resulted in inconsistent sample sizes for each parity group; therefore, we conducted the statistical analysis on the overall sample rather than comparing individual farms.

Positive rate analysis for Kit A indicated that the P0 group showed significantly higher positive rates compared to those of the P1 and P2 groups. The P1 group showed significantly lower positive rates compared to that of the P5+ group. The positive rate of the P2 group did not differ significantly from that of the P1 group but was significantly lower than those of the other parity groups. No significant differences were observed among the remaining parity groups. For Kit B, no significant differences in the positive rates were observed across all the parity groups. For Kit C, the P4 group had significantly lower positive rates compared to that of the P0 group. No significant differences were observed among the other parity groups.

Statistical analysis of the ELISA test values indicated that, for Kit A, the values for the P1 and P2 groups were significantly lower than that for the P0 group. The value for the P2 group was also significantly lower than that of the P5+ group. No significant differences were observed among the remaining parity groups. For Kit C, the value for the P4 group was significantly lower than that for the P0 group. No significant differences were observed among the other parity groups. For Kits B and D, no significant differences were observed across all the parity groups (Figure 4A–D).

The comparison of positive rates across parity groups generally aligned with the comparisons of the mean values, although there were minor discrepancies.

### 3.5. A Negative Result from Kit A Does Not Necessarily Mean the Sample Was Neutralizing Antibody-Negative

**Figure 5:**
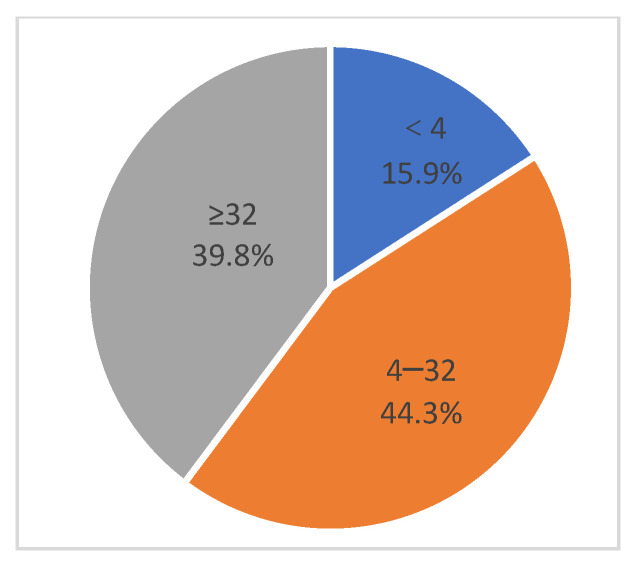
**Distribution of the VNT levels in 88 Kit A-negative samples.** VNT, virus neutralization test.

**Figure 6:**
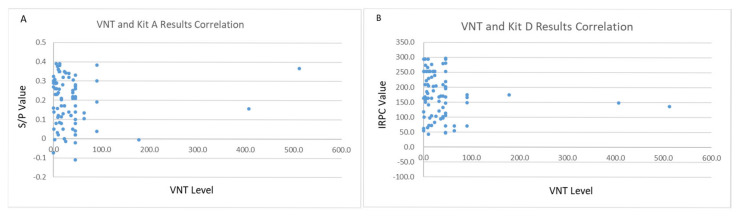
**Correlation between the VNT and the Kit A and Kit D ELISA test results.** (**A**) No correlation was identified between the VNT and Kit A (r = 0.20), where the x-axis indicates the VNT level and the y-axis represents the S/P value. (**B**) No correlation was detected between the VNT and Kit D (r = 0.41), with the x-axis indicating the VNT level and the y-axis representing the IRPC value.

To investigate the true immune status of pigs that tested negative using Kit A, neutralizing antibody tests were conducted on these 88 negative samples. Correlation analysis was performed between the neutralizing antibody test results and the paired Kits A and D test values for the 88 samples (Figure 5 and Figure 6).

Among the 88 Kit A-negative samples, only 15.9% had neutralizing antibody titers less than 4 (2Log^2^), 44.3% had titers between 4 and 32, and 39.8% had titers greater than 32. Statistical analysis revealed that there was no correlation between the VNT and Kit A results (r = 0.20) or the Kit D results (r = 0.41), despite Kit D presenting a more level correlation than Kit A.

## 4. Discussion

PRRS has remained among the top three swine diseases, with high nationwide prevalence, causing huge economics losses to the Chinese swine industry. In 2022, the PRRS-positive rate by farm was 58%, with 74% of pigs being infected before weaning [1]. MLV has been approved frequently as an effective tool to reduce the losses caused by field PRRSV circulation and re-introduction to farms [2]. The Ingelvac PRRS^®^ MLV vaccine has been demonstrated to offer cross-protection against over 10 genetically diverse PRRSV isolates [3,4,5], including several field-isolated NADC30-like strains currently circulating in China [7,8]. In PRRS MLV mass vaccinated herds, the ELISA antibody test is the most commonly used method to monitor the PRRSV immune status because of its rapidity and stability. In the Chinese market, two types of ELISA kits are predominantly used: N-protein-based kits, represented by IDEXX and JNT kits, and GP-protein-based kits, exemplified by ANHEAL and HIPRA kits.

Based on the Herd Immunity Threshold (HIT) formula, when the number of immune individuals reaches (1-1/R0)/VE (vaccine efficacy), the spread of the pathogen within the population ceases, where the R0 value represents the transmission coefficient of the infectious disease. According to the current research, the R0 values of PRRSV are 2.78 and 5.42, respectively, and the homologous protection rate of PRRSV MLV is 87.3% [11,13,18]. Based on these data, the immunization rate against PRRSV needs to reach 73.3 to 93.4% to prevent the dissemination of PRRSV within a population. However, the R0 value of PRRSV is not constant and is related to the strain. Most of the current epidemic PRRSV strains do not belong to the same lineage as the commercial vaccines, thus providing heterologous protection. The homologous protection provided by PRRSV MLV is higher than the heterologous protection [19]. Therefore, the immunization rate to achieve the prevention of virus dissemination within a herd is not a fixed value and is related to the R0 value of the prevalent strain in the farm and the protection rate of the vaccine utilized.

In this study, the ELISA-positive detection rates ranged from 50.0% to 100.0%, depending on different farms using different ELISA kits. Notably, all gilts and sows had received at least two vaccinations before sampling and were maintained at a vaccination frequency of once every 3–4 months. Previous studies have also reported negative ELISA results in pigs from farms vaccinated with a PRRSV MLV vaccine or from farms detected with a field virus. They reported ELISA-negative rates ranging from 1.7% to 30.8% based on different farms using different ELISA kits [6,9,10].

There could be various reasons for animals testing negative using ELISA tests in routinely vaccinated farms. These include issues related to vaccination compliance, such as inadequate herd coverage or vaccination leakage, factors associated with the ELISA kits themselves, or immunological reasons. Two hypotheses from an immunological perspective have been proposed: (1) Repeated vaccination with the same MLV vaccine does not elicit a good humoral response because of a lower antigen level or lack of adjuvant concentration compared to the killed vaccine. (2) Repeated vaccination of pigs with an MLV vaccine might induce lymphocyte anergy [6]. The mechanism of this anergy still needs to be established. It is worth mentioning that a herd does not need to be 100% immunized to prevent the disease from spreading within the herd. Specifically, for heterogenous protection provided by a PRRSV MLV vaccine, an effective vaccine should not only be able to reduce clinical symptoms and production losses but also lower the R0 value of the field virus presented in the farm [11,13].

Normally, PRRS anti-N-protein antibodies are detected from day 7 to 14, and the anti-GP-protein antibodies are initially detected from day 14 to 21 following live virus infection. The duration of the production of antibodies to N-protein and the GP-protein, and neutralizing antibodies could all be sustained over 4 months [15,16,17]. However, this study identified significant variability in the antibody test outcomes across ELISA kits, reflected in differing S/P ratios, IRPC values (CV ranges 37–78%), and inconsistent positive rates (Kit D: 100% vs. Kit A: 60.1%). These findings are comparable with those of previous studies [10,12].

Correlation analysis between the paired values of different kits showed weak to no correlation, with the exception of a strong correlation between Kits A and B (r = 0.80). Overall, the GP-protein-based kits had higher positive rates than the N-protein-based ones, but no significant differences were found between specific N (A vs. B) and GP (C vs. D) kit pairs. The strong correlation and medium Kappa agreement between Kits A and B might reflect the smaller molecular size of the N-protein, which results in higher epitope similarity. Compared with the N-protein, the GP-protein is larger and has a more complicated transmembrane glycoprotein structure. These characteristics might lead to stronger immunological responses and, consequently, a longer antibody duration and higher positive rates [11,20,21]. The complicated multi-epitope structure of the GP-protein might also be the reason why, although Kits C and D are both coated with the GP-protein, they basically showed no correlation in the ELISA test values or Kappa agreement. These discrepancies might be explained as follows: (1) When PRRSV infects target cells, its membrane proteins would first bind to the host cell receptor, followed by identification of these membrane proteins by B cell receptors. This would activate B cells without antigen processing, thereby rapidly triggering an early antibody response. In contrast, nucleocapsid proteins are primarily encapsulated within the virus and require the cell to lyse and release free proteins or be processed by antigen-presenting cells (APCs) before they can be recognized by the immune system [11,20,21]. (2) During virus replication in cells, the N-protein is synthesized earlier than the GP-protein, which would lead to different immunological responses by the host and consequently a lower correlation between the N- and GP-protein antibody test results [22].

Furthermore, our analysis revealed significant differences in the antibody mean values and positive rates across different parities. The P1 and P2 groups showed lower antibody levels, particularly when compared to higher parity groups (P4, P5, and above). However, the antibody response in the P0 group was not significantly lower than that of the higher parity groups. These conclusions are primarily based on the test results for Kit A. Further analysis indicated that the lower positive rates in the P1 and P2 groups were not caused by uneven sample distribution. According to the results of Kit A, among the 41 negative samples from the P1 and P2 groups, although the majority (33 samples) came from farms A and B, with only 8 from farms C and D, the positive rate in the 14 P1 and P2 samples from farms C and D was also low, at about 33%. Additionally, the positive rates for the P0 group in farms A and B reached 82% and 70%, respectively, suggesting that the low antibody levels in the P1 and P2 groups could not be attributed to immunization compliance issues in individual farms. For the lower antibody levels observed in the P1 and P2 groups, we propose the following hypotheses: (1) P1 and P2 parity sows might experience a phenomenon akin to “second litter syndrome” when their physical condition is poorer, which would affect their immune response efficacy, as reported by a previous study [23]. (2) Higher parity sows have experienced more wild virus infections and vaccine immunizations over time, resulting in higher antibody levels. Meanwhile, P0 sows are an exception, probably because they received more intense vaccination during the gilt development period, in addition to the gestation period, as hypothesized in a previous study [12]. Determination of the specific reasons underlying these observations requires further experimental research.

In this study, we have to note that the sampling approach employed was passive surveillance. Specifically, samples from pig farms where issues had been detected were dispatched to Shandong Agricultural University for reconfirmation and further study, and they merely represented a specific cohort. Additionally, the study pertained to a phenomenon within a specific scenario in which the sow herd received regular mass vaccinations and showed stable production. In conclusion, the research data merely represent the test outcomes of each kit in a specific herd under a specific scenario. Thus, the results cannot refute the high sensitivity and high specificity of these kits, particularly Kit A, in the vast majority of scenarios, especially in early detection, compared to Kit D [24,25]. Moreover, Kit A is widely used worldwide and is an international gold standard supported by large amounts of data. However, it cannot be denied that even the gold standard fails to achieve 100% sensitivity and specificity in all circumstances.

For the aforementioned specific groups and scenarios, in the event that the positive rate and S/P value of anti-N-protein antibodies continue to decline, it is advisable to employ the GP-protein antibody kit in combination to determine the immunity level. If feasible, it is recommended to detect the neutralizing antibody level of the group to ascertain the immune status of the herd and consider the neutralizing antibody level as the ultimate approach to determine whether the group still has immunity. Alternatively, an ELISA kit capable of detecting PRRSV-neutralizing antibodies could be developed to address the issue of insufficient ELISA detection sensitivity in this particular scenario.

## 5. Conclusions

The low correlation between different kits suggests that utilizing multiple diagnostic kits, especially combining N with GP ELISA kits, could increase the positive detection rate. Although ELISA is effective to assess PRRSV infection, it is not the optimal tool to measure the immune status because of its poor correlation with neutralizing antibody levels. Sows in their first and second parities (P1 and P2) exhibited lower immune responses. Thus, further research is needed to confirm the specific causes.

## Figures and Tables

**Table 1:** Parity sample distribution.

Farms	Parities						Total
	P0	P1	P2	P3	P4	P5+	
Farm A	20	20	10	10	10	0	70
Farm B	11	14	14	14	0	0	53
Farm C	0	2	9	9	14	6	40
Farm D	20	0	3	15	6	26	70
Total	51	36	36	48	30	32	233

**Table 2:** Comparison of the positive rates for four ELISA kits across farms.

FARMS/KITS	KIT A		KIT B		KIT C		KIT D	
	Pos.% ^1^	S/P ^2^	Pos.%	S/P	Pos.%	S/P	Pos.%	IRPC
FARM A	57.1%	0.51	75.7%	0.66	80.0%	0.59	100.0%	215.1
FARM B	50.9%	0.59	73.6%	0.77	94.3%	0.92	100.0%	216.2
FARM C	50.0%	0.46	70.0%	0.93	82.5%	0.68	100.0%	238
FARM D	75.7%	0.80	75.7%	0.65	97.1%	0.77	100.0%	142.4
RANGE	50.0%, 75.7%	−0.11, 2.20	70.0%, 75.7%	−0.12, 2.44	82.5%, 97.1%	0.03, 2.58	-	43.05 253.30
AVERAGE	58.4%	0.61	73.7%	0.76	88.5%	0.75	100.0%	197.45
95% CI ^3^	48.4%, 68.4%	0.55, 0.67	71.2%, 76.0%	0.70, 0.83	81.2%, 95.2%	0.70, 0.80	100.0%, 100.0%	187.90, 207.00
CV	-	78%	-	66%	-	50%	-	37%
TOTAL	60.1%	-	74.2%	-	88.8%	-	100.0%	-

Note: ^1^. In the “Pos.%” column, the value for each farm indicates the positive rate of that farm. ^2^. In the “S/P” column, the value for each farm indicates the mean S/P value for that farm. ^3^. “95% CI” refers to the 95% confidence interval of the average positive rate. Underlined numbers indicate the largest value among Kits A–D.

**Table 3:** Kappa efficiency between the pairs of ELISA kits.

Kappa = 0.59		Kit A		Total	Kappa = 0.16		Kit A		Total
		Positive	Negative				Positive	Negative	
Kit B	Positive	135	38	173	Kit C	Positive	132	75	207
	Negative	5	55	60		Negative	8	18	26
Subtotal		140	93	233	Subtotal		140	93	233
Kappa = 0		Kit A		Total	Kappa = 0.17		Kit B		Total
		Positive	Negative				Positive	Negative	
Kit D	Positive	140	93	233	Kit C	Positive	160	47	207
	Negative	0	0	0		Negative	13	13	26
Subtotal		140	93	233	Subtotal		173	60	233
Kappa = 0		Kit B		Total	Kappa = 0		Kit D		Total
		Positive	Negative				Positive	Negative	
Kit D	Positive	173	60	233	Kit C	Positive	207	0	207
	Negative	0	0	0		Negative	26	0	26
Subtotal		173	60	233	Subtotal		233	0	233

**Table 4:** Positive rates of the total samples by parity and kits.

Parity	P0	P1	P2	P3	P4	P5+
Sample Size	51	36	36	48	30	32
Kit A	75%	50%	31%	58%	67%	71%
Kit B	82%	72%	64%	67%	80%	86%
Kit C	96%	83%	86%	92%	77%	100%
Kit D	100%	100%	100%	100%	100%	100%

## Data Availability

The data utilized in this study are not publicly available due to privacy restrictions.

## Abbreviations

The following abbreviations are used in this manuscript:

PRRSV	Porcine reproductive and respiratory syndrome virus
MLV	Modified live virus
N protein	Nucleocapsid protein
GP protein	Glycoprotein
VNT	Viral neutralizing test
VN	Viral neutralization
ELISA	Enzyme-linked immunosorbent assay
ASF	African swine fever
IRPC	Relative Index Percentage
CPE	Cytopathic effects
